# A scientific report on heat transfer analysis in mixed convection flow of Maxwell fluid over an oscillating vertical plate

**DOI:** 10.1038/srep40147

**Published:** 2017-03-15

**Authors:** Ilyas Khan, Nehad Ali Shah, L. C. C. Dennis

**Affiliations:** 1 Basic Engineering Sciences Department, College of Engineering Majmaah University, P.O. Box 66, Majmaah 11952, Saudi Arabia; 2 Abdus Salam School of Mathematical Sciences, GC University, Lahore, Pakistan; 3 Department of Fundamental and Applied Sciences, Universiti Teknologi PETRONAS, 31750 Perak, Malaysia

## Abstract

This scientific report investigates the heat transfer analysis in mixed convection flow of Maxwell fluid over an oscillating vertical plate with constant wall temperature. The problem is modelled in terms of coupled partial differential equations with initial and boundary conditions. Some suitable non-dimensional variables are introduced in order to transform the governing problem into dimensionless form. The resulting problem is solved via Laplace transform method and exact solutions for velocity, shear stress and temperature are obtained. These solutions are greatly influenced with the variation of embedded parameters which include the Prandtl number and Grashof number for various times. In the absence of free convection, the corresponding solutions representing the mechanical part of velocity reduced to the well known solutions in the literature. The total velocity is presented as a sum of both cosine and sine velocities. The unsteady velocity in each case is arranged in the form of transient and post transient parts. It is found that the post transient parts are independent of time. The solutions corresponding to Newtonian fluids are recovered as a special case and comparison between Newtonian fluid and Maxwell fluid is shown graphically.

Exact solutions for mixed or free convection flow of viscous fluid problems are abundance in literature. However, such solutions for non-Newtonian fluids are rare, particularly for Maxwell fluids, such solutions do not exist. Generally, in non-Newtonian fluids, the relation which connects shear stress and shear rate is non-linear and the constitutive relation forms equations of non-Newtonian fluids which are higher order and complex as compared to Navier-Stokes equation governing the flow of viscous fluid. Due to this high nonlinearity, closed form solutions for non-Newtonian fluid flows are not possible for the problems with practical interest. More exactly, when such fluids problems are tackled via Laplace transform technique, often the inverse Laplace transforms of the transformed functions do not exist. Due to this difficulty, the researchers are usually using numerical procedures for finding the inverse Laplace transform. However, those solutions are not purely regarded as exact solutions.

Due to the great diversity in the physical structure of non-Newtonian fluids, researchers have proposed a variety of mathematical models to understand the dynamics of such fluids. Mostly, these models fall in the subcategory of differential type fluids or rate types fluids. However, a keen interest of the researchers is seen in studying rate types fluids due to the fact that they incorporate both the elastic and memory effects together. The first and the simplest viscoelastic rate type model which is still used widely to account for fluid rheological effects is called Maxwell model^1^. This model can be generalized to produce a plethora of models. Initially, the Maxwell fluid model was developed to describe the elastic and viscous response of air. However, after that, it was frequently used to model the response of various viscoelastic fluids ranging from polymers to the earth’s mantle^2^,^3^,^4^. After the pioneering work of Friedrich^5^, on fractional derivatives of Maxwell fluid, several other investigations were carried out in this direction.

Among them,Haitao and Mingyu^6^ studied fractional Maxwell model in channel, Jamil *et al*.^7^ analyzed unsteady flow of generalized Maxwell fluid between two cylinders. In another investigation, Jamil *et al*.^8^, examined helices of fractionalized Maxwell fluid whereas Jamil^9^ analyzed slip effects on oscillating fractionalized Maxwell fluid. Corina *et al*.^10^ provided a short note on the second problem of Stokes for Maxwell fluids. Zheng *et al*.^11^, developed exact solutions for generalized Maxwell fluid for oscillatory and constantly accelerating plate motions, Zheng *et al*.^12^ used the same fluid model for heat transfer study due to a hyperbolic sine accelerating plate. Qi and Liu^13^ studied some duct flows of a fractional Maxwell fluid. Tripathi^14^ applied fractional Maxwell model to study peristaltic transport in uniform tubes.

Fetecau and Fetecau^15^, established a new exact solution for the flow of a Maxwell fluid past an infinite plate. In an other investigation, Fetecau and Fetecau^16^ determined exact solutions by means of the Fourier sine transforms for an incompressible fluid of Maxwellian type subjected to a linear flow on an infinite flat plate and within an infinite edge. Jordan *et al*.^17^ studied Stokes’s first problem for Maxwell fluids and obtained new exact solutions. Zierep and Fetecau^18^ examined energetic balance for the Rayleigh-Stokes problem of Maxwell fluid. Among some other important studies on Maxwell fluids, we mention here the important contributions of Jamil *et al*.^19^, Vieru and Rauf^20^, Vieru and Zafar^21^ and Khan *et al*.^22^. However, in all these investigations, heat transfer analysis was not considered. More exactly, phenomenon of heat transfer due to mixed convection was not incorporated in all the above studies. Therefore, the focal point of this work is to analyze Maxwell fluid over an oscillating vertical plate with constant wall temperature and to establish exact solutions using the Laplace transform method. The obtained results consideration of heat transfer analysis in Maxwell fluid has industrial importance since many problems of physical interest involve heat transfer such as automotive industry (radiator, cooling circuits, lamps), aerospace (de-icing system, cooling systems), in chemical process industry (heat recovery systems, heat exchangers), energy (kilns, boiler, cross flow heat exchangers, solar panels) and home appliance (ovens, household heaters)^23^,^24^,^25^.

## Mathematical formulation of the problem

Let us consider unsteady mixed convection flow of an incompressible Maxwell fluid over an oscillating vertical flat plate moving with oscillating velocity in its own plane. Initially, at time *t* = 0, both the fluid and the plate are at rest with constant temperature *T*_∞_. At time *t* = 0^+^ the plate is subjected to sinusoidal oscillations so that the velocity on the wall is given by V = *U*_*0*_*H(t*)cos(ω*t*), resulting in the induced Maxwell fluid flow. More exactly, the plate begins to oscillate in its plane (*y* = 0) according to **V** = *U*_*0*_*H(t*)cos(ω*t*)**i**; where the constant *U*_0_ is the amplitude of the motion, *H(t*) is the unit step function, **i** is the unit vector in the vertical flow direction and *ω* is the frequency of oscillation of the plate. At the same time *t* = 0^+^, the temperature of the plate is raised or lowered to a constant value *T*_*w*_. The velocity decays to zero and temperature approaches to a constant value *T*_∞_, also known as free stream temperature. The equations governing the Maxwell fluid flow related with shear stress and heat transfer due to mixed convection are given by the following partial differential equations:


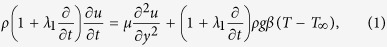



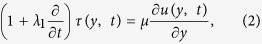



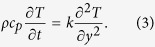


The appropriate initial and boundary conditions are:


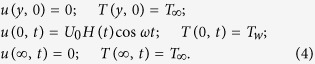


Introducing the following non-dimensional quantities:


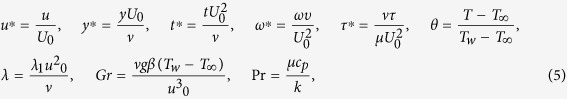


into Eqs (1, 2, 3), we get


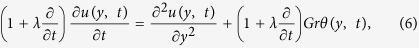



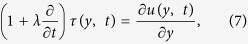



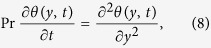


with the corresponding initial and boundary conditions:


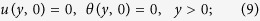



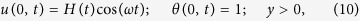



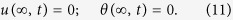


## Solution of the problem

### Temperature

Taking Laplace transform of Eqs (8), (10)_2_, (11)_2_ and using initial condition (9)_2_, we obtain


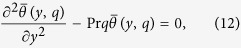



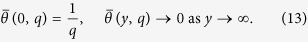


The solution of the partial differential equation (12) subject to conditions (13) is given as:


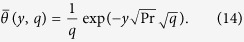


Taking the inverse Laplace transform and using (A1), we obtain:


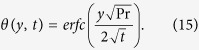


### Velocity field

Taking the Laplace transform of Eqs (6), (10)_1_, (11)_1_ and using initial conditions, we obtain


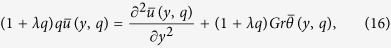



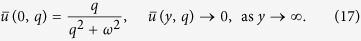


Using Eq. (14) in Eq. (16), we have


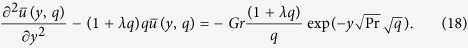


Solve the partial differential Eq. (18), we have:


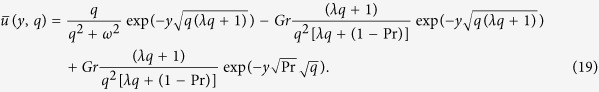


The last equality can be written in equivalent form as:


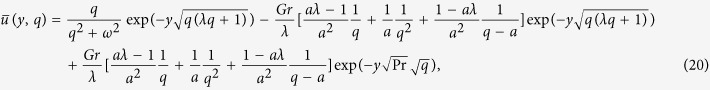


where 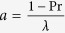.

Let


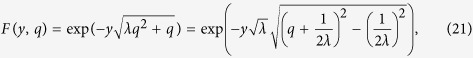



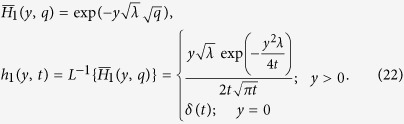


Taking the inverse Laplace transform of Eq. (21), we obtain:


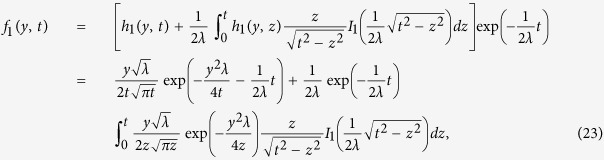



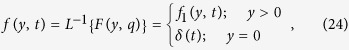



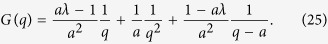


Taking the inverse Laplace of Eq. (25), we obtain


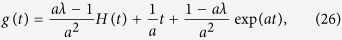



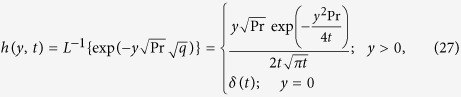


where 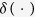 being Dirac distribution.

Applying inverse Laplace transform to Eq. (20) and using convolution product, we obtain


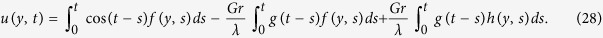


### Shear stress

Applying Laplace transform to Eq. (7), we obtain


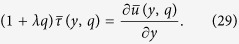


Differentiate Eq. (19) with respect to spatial variable 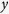, we obtain


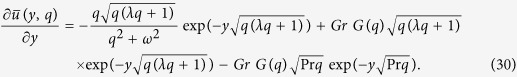


Put Eq. (30) into Eq. (29), we obtain


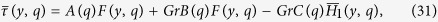


where


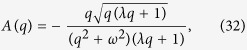



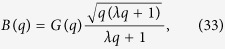



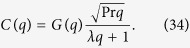


Applying the inverse Laplace transform to Eqs (31), (32), (33) and (34), we obtain


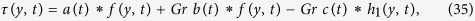


with


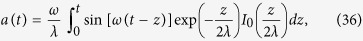



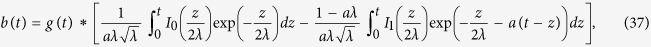



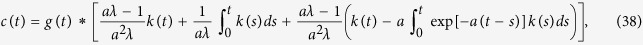


where 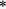 represents convolution product and 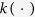 is defined in Appendix (A3).

## Solutions in the absence of Buoyancy force (limiting case)

In this case, when *Gr* = 0 the solution corresponding to oscillating boundary motion can easily be obtained from Eqs. (28) and (35). Such solutions are already obtained by Corina *et al*.^10^.

### Newtonian fluid (*λ* = 0).

#### Velocity


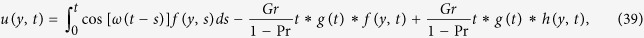



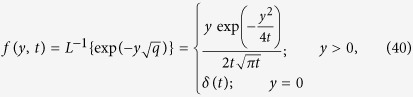



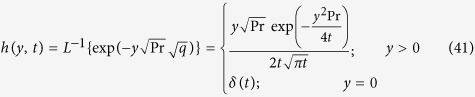


#### Shear stress


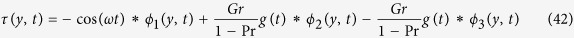


where


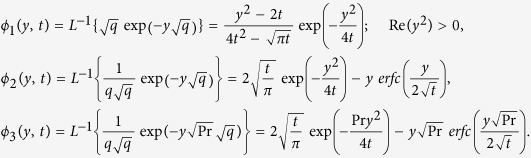


## Numerical results and discussions

**Figure 1:**
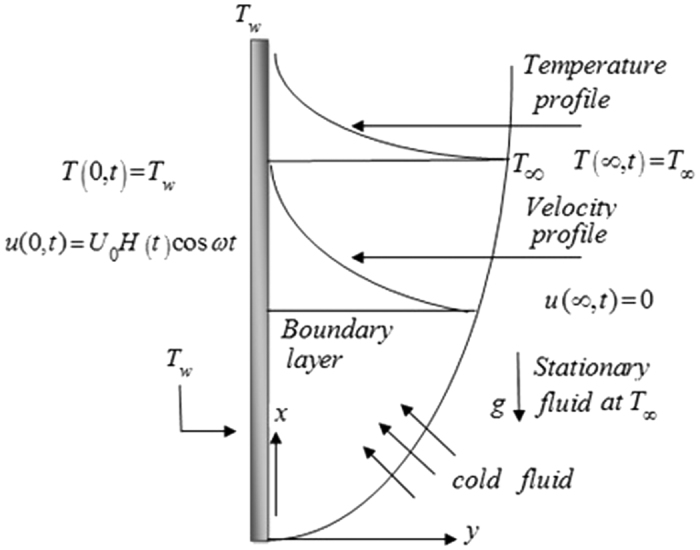
Velocity and temperature profiles for mixed convection flow over a hot vertical plate at *T*_*w*_ exposed to plate at *T*_∞_.

**Figure 2:**
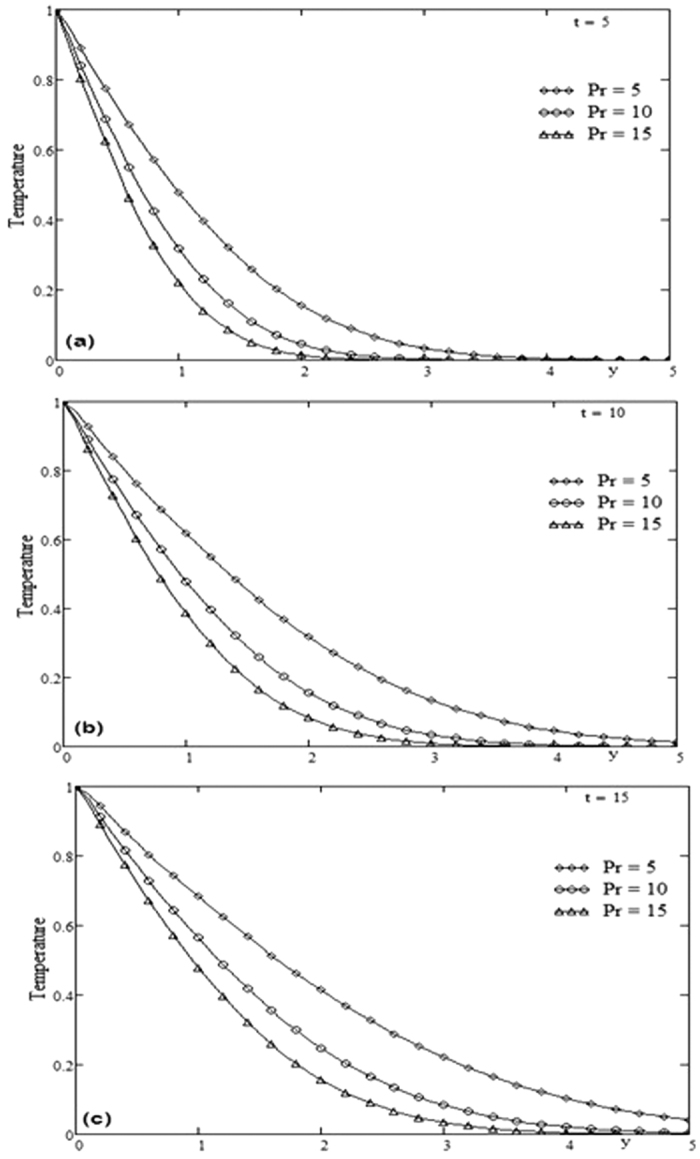
Profiles of temperature for Prandtl number Pr variation for different time t.

**Figure 3:**
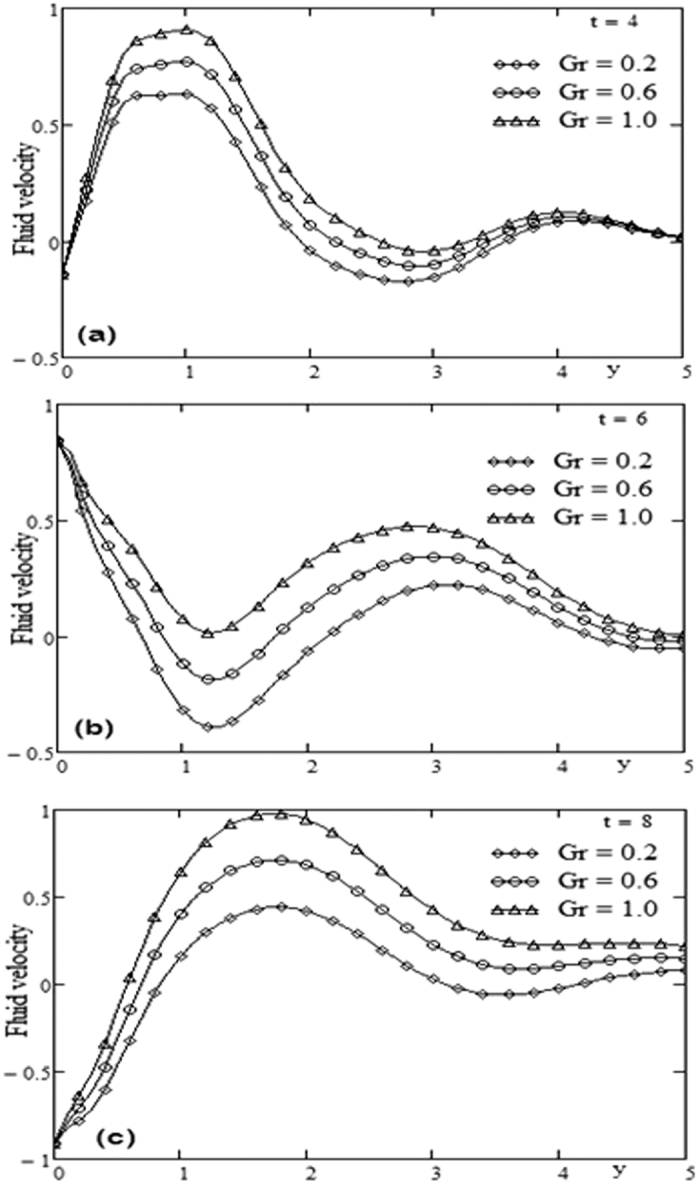
Profiles of velocity for Grashof number Gr variation for different time t.

**Figure 4:**
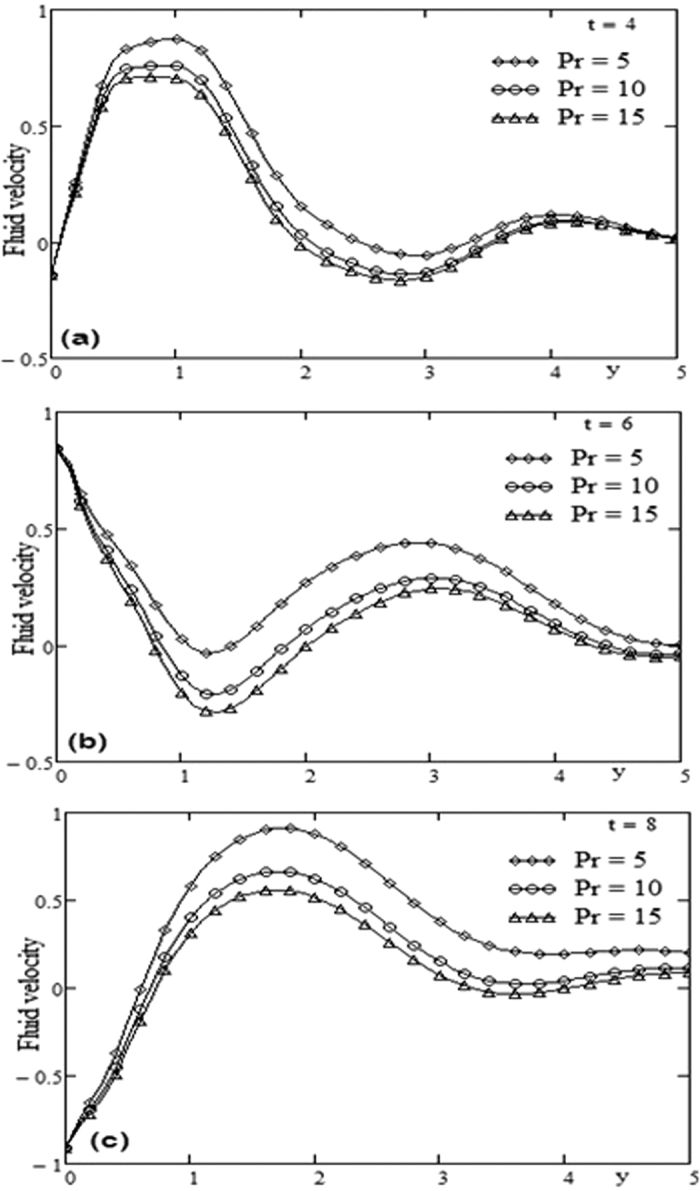
Profiles of velocity for Prandtl number Pr variation for different time t.

**Figure 5:**
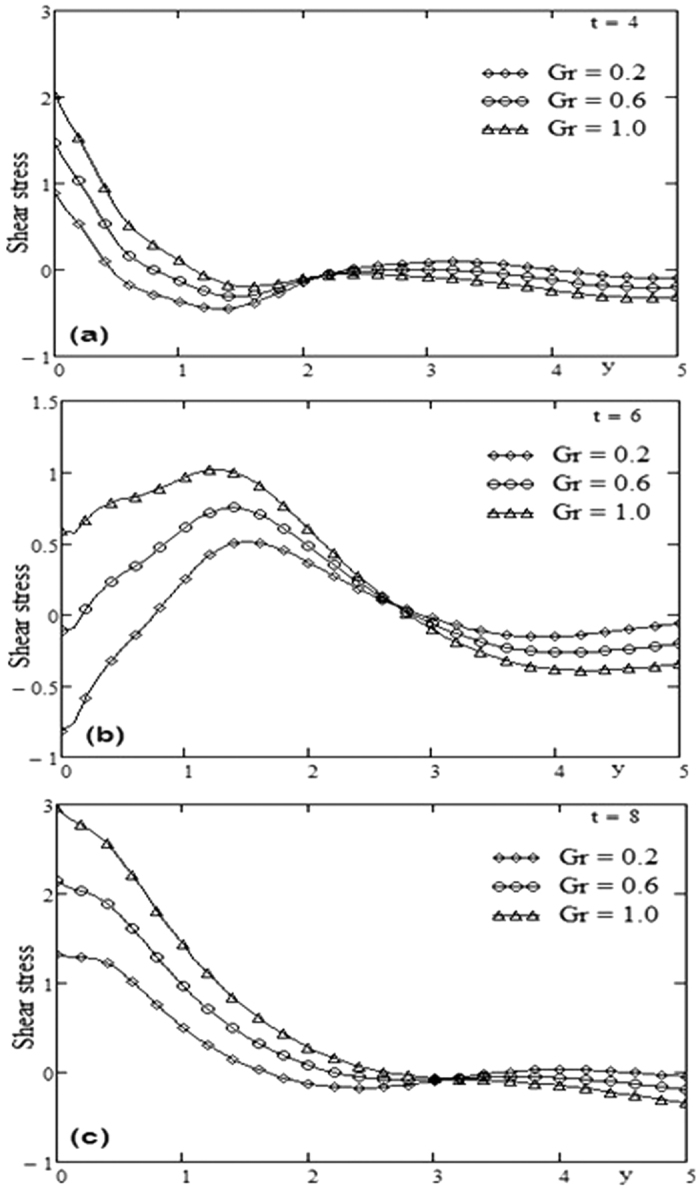
Profiles of shear stress for Grashof number Gr variation for different time t.

**Figure 6:**
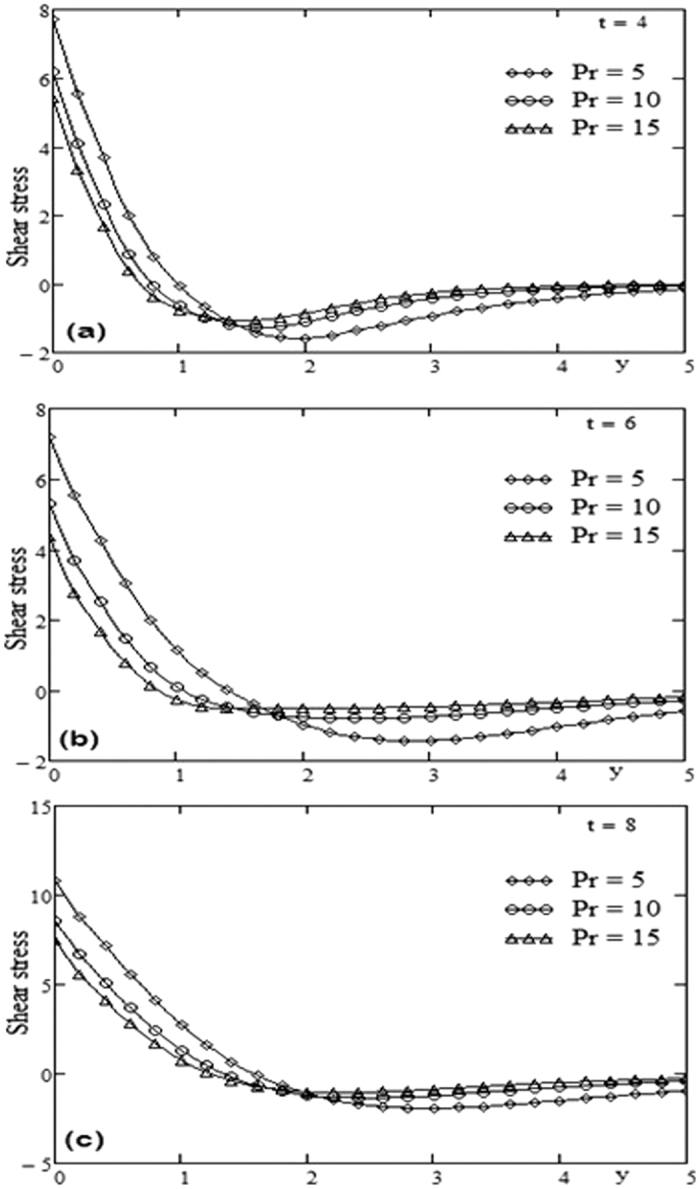
Profiles of shear stress for Prandtl number Pr variation for different time t.

**Figure 7:**
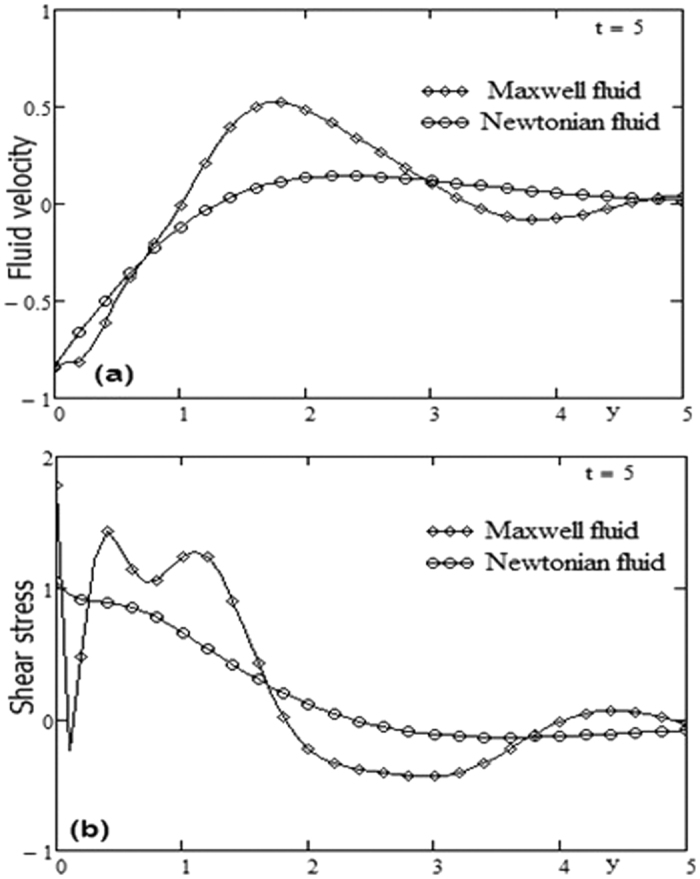
Profiles of velocities and shear stress for Maxwell fluid and Newtonian fluid.

The geometry of the problem is given in Fig. 1. In order to get some physical insight of the results corresponding to oscillating velocity on the boundary, some numerical calculations have been carried out for different values of pertinent parameters that describe the flow characteristics. All physical quantities and profiles are dimensionless. Also all profiles are plotted versus *y*. Figure 2 presents the temperature profiles for different values of time *t* and Prandtl number Pr variation. The fluid temperature is a decreasing function with respect to Prandtl number Pr and tends to a steady state slowly as the time *t* increases. Figure 3 presents the velocity profiles for different values of time *t* and Grashof number *Gr* variation. For other constant we have *λ* = 0.7, ω = 2, Pr = 5. It is observed that the fluid velocity is increased by increasing the Grashof number *Gr*. By increasing the time *t* the difference between the velocities as well as the steady state increases. Figure 4 presents the velocity profiles for different values of time *t* and Prandtl number Pr variation. For other constants, we have *λ* = 0.7, ω = 2, *Gr* = 5. It is observed that the fluid velocity decreases by increasing the Prandtl number Pr. By increasing the time *t*, the difference between the velocities as well as the steady state increases. Figure 5 presents the shear stress profiles for different values of time *t* and Grashof number *Gr* variation. For other constants, we have 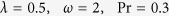. It is observed that near the boundary the shear stress increases by increasing the Grashof number *Gr* but after some critical value of *y* the shear stress is decreased by increasing *Gr*. By increasing the time *t* the critical value of *y* is increased it means that the critical point is far from the boundary. Figure 4 presents the shear stress profiles for different values of time and Prandtl number Pr variation. For other constants we have *λ* = 0.3, ω = 2, *Gr* = 10. It is observed that the region near the boundary, the shear stress is decreased by increasing the Prandtl number Pr. By increasing the time *t*, Fig. 6 has the same behavior like Fig. 4. A comparison between Maxwell fluid and Newtonian fluid is shown graphically in Fig. 7.

## Conclusions

This study reports the first exact solution for unsteady mixed convection problem of Maxwell fluid via Laplace transform method. Expressions of velocity, shear stress and temperature are obtained and then plotted graphically for various embedded parameters. The solution corresponding to Newtonian fluid problem is recovered as a special case. Moreover, it is found that in the absence of free convection term, the already published results can be recovered as a special case. From the plotted results, it is found that temperature decreases with increasing Prandtl number; however, for large timethe temperature decays later. Velocity decreases with increasing Prandtl number whereas an oscillating behavior is observed for Grashof number.

## Additional Information

**How to cite this article:** Khan, I. and Shah, N. A. A scientific report on heat transfer analysis in mixed convection flow of Maxwell fluid over an oscillating vertical plate. *Sci. Rep.* **6**, 40147; doi: 10.1038/srep40147 (2016).

**Publisher's note:** Springer Nature remains neutral with regard to jurisdictional claims in published maps and institutional affiliations.

## Supplementary Material

Supplementary Information
