# Erratum: A scientific report on heat transfer analysis in mixed convection flow of Maxwell fluid over an oscillating vertical plate

**DOI:** 10.1038/srep46975

**Published:** 2018-05-17

**Authors:** Ilyas Khan, Nehad Ali Shah, L. C. C. Dennis

Scientific Reports 7: Article number: 4014710.1038/srep40147; published online: 03 15 2017; updated: 05 17 2018

In the original version of this Article, the author L. C. C. Dennis was inadvertently omitted from the Author Contributions statement and the Supplementary Information.

The Author Contributions statement,

“I.K. formulated the problem N.A.S. solved the problem and prepared the figures. I.K. and N.A.S. wrote the main manuscript text. Both authors reviewed the manuscript.”

now reads:

“I.K. formulated the problem. N.A.S. solved the problem. L. C. C. Dennis performed the numerical simulations and prepared the results. I.K. and N.A.S. wrote the physical discussion of the figures. I.K, and N.A.S wrote main manuscript text. L. C. C. Dennis proof read the manuscript.”

In addition, the Acknowledgements section was omitted and now reads:

“The authors acknowledge with thanks the Deanship of Scientific Research (DSR) at Majmaah University, Majmaah Saudi Arabia for technical and financial support through vote number 37/97 for this research project.”

These errors have now been corrected in the HTML and PDF versions of the Article, and in the accompanying Supplementary Information.

