# Supplementary material for: A scientific report on heat transfer analysis in mixed convection flow of Maxwell fluid over an oscillating vertical plate
Source: Sci Rep. 2017 Mar 15;7:40147. doi: 10.1038/srep40147 (PMC5353556; doi:10.1038/srep40147)
Supplement: Supplementary Information [file srep40147-s1.doc]

**A scientific report on heat transfer analysis in mixed convection flow of Maxwell fluid over an oscillating vertical plate**

Ilyas Khan^1^ and Nehad Ali Shah^2^,

^1^Basic Engineering Sciences Department, College of Engineering Majmaah University, P.O. Box 66, Majmaah 11952, Saudi Arabia.

^2^Abdus Salam School of Mathematical Sciences, GC University, Lahore, Pakistan.

^*^Address corresponding to Ilyas Khan: [i.said@mu.edu.sa/](mailto:i.said@mu.edu.sa/) ilyaskhanqau@yahoo.com

**Appendix**

$L^{-1}\left\{\frac{\exp\left(-c\sqrt{q+b}\right)}{q-a}\right\}=\frac{\exp(at)}{2}\left[\begin{matrix} \exp\left(-c\sqrt{a+b}\right)erfc\left(\frac{c}{2\sqrt{t}}-\sqrt{(a+b)t}\right)+ \\ +\exp\left(c\sqrt{a+b}\right)erfc\left(\frac{c}{2\sqrt{t}}+\sqrt{(a+b)t}\right) \end{matrix}\right].$ (A1)

$L^{-1}\left\{e^{-y\sqrt{a}\sqrt{q}}\right\}=\frac{y\sqrt{a}e^{-\frac{y^{2}a}{4t}}}{2t\sqrt{\pi t}}$ (A2)

$k(t)=L^{-1}\left\{\frac{1}{\sqrt{q}\left(\lambda q+1\right)}\right\}=\frac{1}{\sqrt{\pi\lambda}}\int_{0}^{\infty} \sin\left(\frac{s}{\lambda}\right)\exp\left(-\frac{s^{2}}{4t}\right)ds.$ (A3)

$L^{-1}\left\{\sqrt{q}\exp\left(-a\sqrt{q}\right)\right\}=\frac{a^{2}-2t}{4t^{2}-\sqrt{\pi t}}\exp\left(-\frac{a^{2}}{4t}\right); \mathrm{Re}(a^{2})>0.$ (A4)

$L^{-1}\left\{\frac{1}{q\sqrt{q}}\exp\left(-a\sqrt{q}\right)\right\}=2\sqrt{\frac{t}{\pi}}\exp\left(-\frac{a^{2}}{4t}\right)-a erfc\left(\frac{a}{a\sqrt{t}}\right).$ (A5)
